# Are Digital Oral Thermometer Readings Accurate in Adult Emergency Department Patients?

**DOI:** 10.7759/cureus.22047

**Published:** 2022-02-09

**Authors:** Ryan J Reece, Mary Hughes

**Affiliations:** 1 Emergency Medicine, Michigan State University, Flint, USA; 2 Osteopathic Medical Specialties, Michigan State University, Lansing, USA

**Keywords:** severe sepsis, infection rates, comprehensive physical exam, fever of unkown, quality initiatives

## Abstract

Background

Inaccurate vital signs may lead to inadequate treatment and skew the differential diagnosis in patients presenting to the emergency department (ED), and thus could cause a delay in diagnosis and treatment. Our study sought to evaluate and compare oral and rectal temperatures in patients with medical conditions that may have fever as part of their presentation to the ED.

Objectives

To determine if oral and rectal temperatures correlate in patients with medical conditions who have a fever, dry mucous membranes, and are warm to the touch on exam. To identify which patient presentations are more likely to have incongruous temperatures. Our hypothesis is that digital oral thermometers are inaccurate and understate the temperature in patients who present with dry mucous membranes and tactile warmth.

Methods

A prospective cohort of adult patients in the ED was asked to consent to the comparison of rectal temperature if they presented with a medical condition that could result in a fever. Oral and rectal (core) temperatures were obtained, along with demographic data, chief complaint, current medications, recent ingestion of warm or cold liquids, use of antipyretics, and the treating physician’s assessment of mucous membrane dryness and tactile warmth.

Results

A total of 111 patients were enrolled in the study. 55.8% of patients were male, and the mean age was 61 years. The most common presenting complaint was lower respiratory tract related; 87% had dry mucous membranes, and 85.5% were warm to the touch. Fever or hypothermia was missed in 55 patients (49.5%) if only oral temperatures were obtained. Patients were more likely to consent if their doctor was concerned about a fever and requested a rectal temperature as part of their workup.

Conclusions

This is one of the first studies to evaluate the difference between oral and rectal routes of obtaining body temperature in the ED in adult patients. Our data reveal that many fevers are “missed” if only oral temperatures are used in medical decision-making in patients with dry mouths and with tactile warmth. Our study is limited by the small sample size and the potential for selection bias.

## Introduction

Anecdotally, there are many instances of identifying missed fevers by utilizing core temperatures instead of oral temperatures. Physicians need accurate patient data to make accurate decisions regarding patient care. There is a need for objective data to back up the claim that there are missed fevers. Patient management and treatment courses may be altered by the presence of fever [1]. Examples of this include encephalopathy, chest pain, and dyspnea. In 1946, Hales et al. found that “…mouth temperature readings are liable to be fallacious and misleading in acute disease, and that rectal figures afford invaluable clinical evidence in both acute and chronic conditions” [2]. Also, in 1954, Strydom et al. found that in normal healthy volunteers, the average difference between oral and rectal temperatures was 1.22 ℉ [3]. Lastly, Varney et al. published, “A comparison of oral, tympanic, and rectal temperature measurement in the elderly” [4] in 2002. They conducted a cross-sectional study on 95 patients aged 60 and above. A fever is defined as a temperature of more than 100.4 ℉. Fever was missed in 14 of 95 (14.7%) patients if only oral/tympanic temperatures were used. This was the only study that only looked at older adults and was related to emergency department (ED) patients. In comparison, our study is the only one that looked at differences in oral and rectal temperatures in all adult ED patients.

## Materials and methods

Study question and hypothesis

Do oral and rectal temperatures correlate in patients presenting with medical conditions that may have a fever, have dry mucus membranes, and are warm to the touch on an exam?

Which patient presentations are more likely to have incongruous temperatures?

Our Hypothesis

Digital oral thermometers are inaccurate and understate the temperature in patients who present with dry mucous membranes and tactile warmth.

Project design

This study was approved by the Michigan State University Institutional Review Board (MSU IRB Approval #: 16-1378). The prospective cohort of adult patients from January 2017 to March 2018 was asked to consent to a comparison of their core temperature to their oral temperature if they presented with a medical condition that may have caused a fever. Oral and rectal temperatures were obtained via the SureTempTM Digital Thermometer. We also collected demographic data, chief complaints, current medications, ingestion of warm or cold liquids, use of antipyretics, and assessment of oral mucosa and tactile warmth.

In our study, we defined a “missed fever” as an oral temperature of 100.3 ℉ or less with a rectal temperature that was 100.4 ℉ or more in a patient. The thermometer used automatically deducts 1 ℉ from a rectal temperature reading to ensure that the readings from the oral and rectal routes are the same temperature.

Adult (18+ years old) patients who presented to the ED with a condition that could include a fever were included in the study. The exclusion criteria included children, prisoners, persons in custody, isolated musculoskeletal injuries not due to syncope or other general medical problems, trauma activations, subjects on chemotherapy for oncologic or autoimmune disorders, subjects suspected of having neutropenia, subjects without a rectum, subjects with trauma or surgery on their rectum in the last six weeks, and subjects who refuse a rectal temperature.

**Figure 1:**
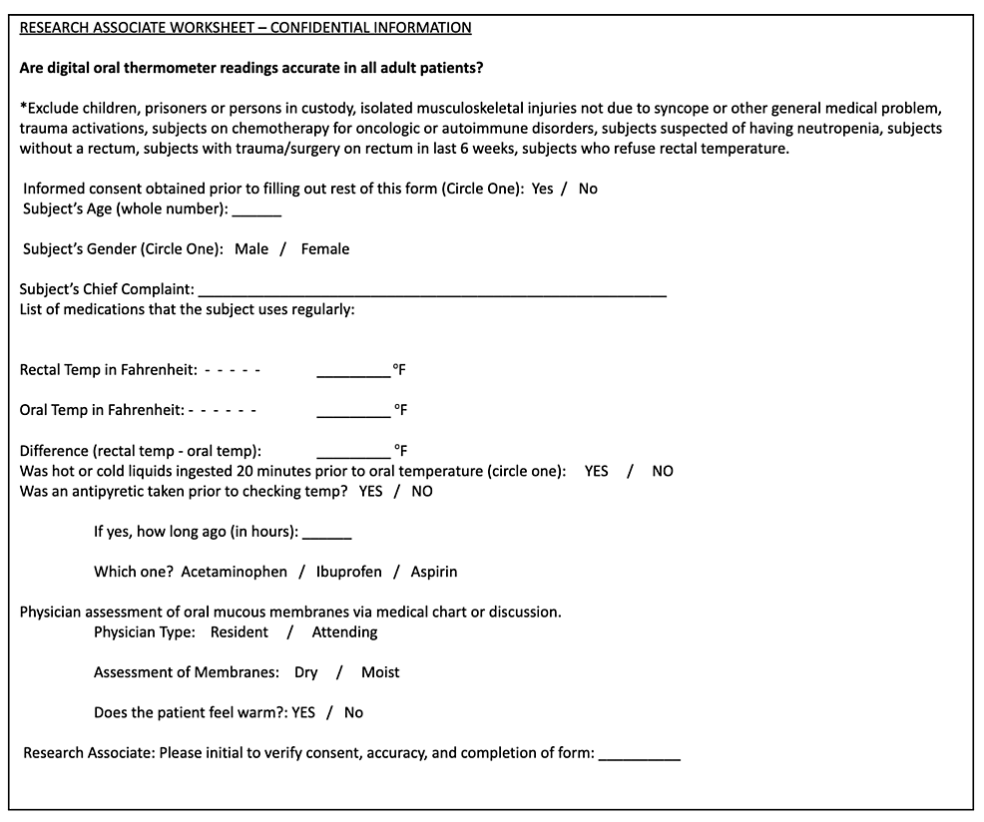
Form used to collect data from subjects

The research staff used the research associated worksheet to collect the necessary data points as seen in Figure 1.

Analysis

SPSS (IBM Corp. Released 2017. IBM SPSS Statistics for Windows, Version 25.0. Armonk, NY, USA) was used to analyze the data. Frequencies were tabulated and analyzed for sensitivities, specificities, negative predictive value, positive predictive value, and applied the chi-square test.

## Results

We enrolled 111 patients from January 2017 to March 2018 in the study; the range of ages was 19-97 years old, with an average age of 61. Of these, 62 were male 47 were female subjects, and 2 of the subjects had missing values for their gender. The rectal temperature average was 100.8 ℉, with a range of 95.5 ℉ to 105.5 ℉. The average oral temperature was 98.9; with a range of 97 ℉ to 103 ℉. Fever was missed in 55 (49.5%) of patients. The most common chief complaints were: 23% lower respiratory, 18% of gastrointestinal and nervous system complaints, and 14% of fever/chills.

Table 1 outlines the distribution of missed fevers by gender. We utilized Chi-Square analysis with a result of 0.440; no statistical significance was noted here.

**Table 1:** Distribution of missed fevers by gender Male % Missed: 46.7; Female % Missed: 53.2

	Fever Missed	
	Yes	No
Male	29 patients	33 patients
Female	25 patients	22 patients

Table 2 outlines the distribution of missed fevers and the clinician's assessment of the tactile warmth of the patient. Sensitivity was 96.3%, specificity was 25.5%, positive predictive value was 55.9%, negative predictive value was 87.5%, and the chi-square test resulted as 0.004.

**Table 2:** Distribution of missed fevers and their associated assessments of tactile warmth

		Fever Missed		
		Yes	No	Totals
Tactile Warmth	Yes	52 patients	41 patients	93 patients
	No	2 patients	14 patients	16 patients
	Totals	54 patients	55 patients	109 patients

Table 3 outlines the distribution of missed fevers when compared to the clinician's assessment of the patient's oral mucosa moisture. Sensitivity was 94.3%, specificity was 19.6%, positive predictive value was 52.6%, negative predictive value was 78.5%, and the chi-square yielded 0.05. 

**Table 3:** Distribution of missed fevers in relation to their respective oral mucosa assessments

		Fever Missed		
		Yes	No	Totals
Oral Mucosa	Dry	50 patients	45 patients	95 patients
	Moist	3 patients	11 patients	14 patients
	Totals	53 patients	56 patients	109 patients

## Discussion

This study is the first of its kind to investigate both objective data by way of patient temperatures as well as subjective data from the treating physician's physical examination. Our results suggest that the physical examination is very sensitive to discovering a possible missed fever. Digital oral thermometers are inaccurate and understate the temperature in patients that present with dry mucous membranes and tactile warmth.

Our study was limited by its small size; we had 111 patients enrolled in our study. It was also limited by possible selection bias; patients with a chief complaint of fever, dysuria, or cough were most likely enrolled in the study and had a core temperature obtained. Another element that may limit the study is that there was no standardized method to obtain an oral temperature. Our study was limited as a few elements were omitted from the data collection sheets inadvertently by the research assistant staff.

The need to find a reliable and accurate method to obtain a patient's temperature has been a priority within the medical community for decades. There is a breadth of knowledge from the literature concerning this topic, however, with mixed results. Shann et al. found that rectal temperatures were similar to axillary temperatures and that forehead temperatures were not accurate [5]. However, Batra et al. found that temporal artery thermometer readings were similar to rectal thermometer readings [6]. Furthermore, Nygaard et al. and Brosinski et al. found that temporal artery thermometer readings were not accurate when compared to rectal thermometer readings [7,8].

This study adds to the medical literature by iterating that the treating clinician's physical examination is important when deciding which temperature route will yield accurate results. Having accurate patient data can help the treating clinician make the right diagnosis and start the correct treatment.

## Conclusions

Based on our data and our definition of a “missed fever,” 49.5% of patients sampled had a fever missed. From our results, oral temperature readings are inaccurate in patients that have dry oral mucosa and have tactile warmth. Instead, obtain a rectal temperature as we found this route to be more sensitive at detecting a fever. The treating clinician should keep the patient’s physical exam in mind when choosing the route of temperature measurement, as well as having an elevated clinical suspicion of a false temperature reading when the patient has a dry mouth and has tactile warmth. Patient care and treatment plans may be altered by the presence or absence of a fever. Having inaccurate physical examination information can lead to poor outcomes if signs of disease, such as fever, are missed.
